# Direct oral anticoagulants for the treatment and prevention of venous thromboembolism in patients with cancer: current evidence

**DOI:** 10.1007/s12094-020-02506-4

**Published:** 2020-11-18

**Authors:** I. García-Escobar, E. Brozos-Vázquez, D. Gutierrez Abad, V. Martínez-Marín, V. Pachón, A. J. Muñoz Martín, Cancer and Thrombosis Section of the Spanish Society of Medical Oncology (SEOM)

**Affiliations:** 1 Servicio de Oncología, Hospital General Universitario de Ciudad Real, Calle Obispo Rafael Torija, s/n, 13005 Ciudad Real, Spain; 2 Servicio de Oncología Médica, Complejo Hospitalario Universitario de Santiago de Compostela, Santiago de Compostela, Spain; 3 Servicio de Oncología, Hospital Universitario de Fuenlabrada, Fuenlabrada, Spain; 4 Servicio de Oncología Médica, Hospital Universitario La Paz, Madrid, Spain; 5 Servicio de Oncología Médica, Hospital Universitario Ramon Y Cajal, IRYCIS, CIBERONC, Madrid, Spain; 6 Servicio de Oncología Médica, Hospital General Universitario Gregorio Marañón, Universidad Complutense, Madrid, Spain

**Keywords:** Venous thromboembolic disease, Cancer, Direct oral anticoagulant agents, Efficacy, Safety

## Abstract

Venous thromboembolic disease (VTED) is a common and clinically important complication in patients with cancer, contributing to its mortality and morbidity. Direct oral anticoagulant agents (DOACs), including direct thrombin inhibitors and direct factor Xa inhibitors, are as effective as vitamin K antagonists for the treatment of VTED and are associated with less frequent and severe bleeding. They have advantages over low-molecular-weight heparin, but comparative long-term efficacy and safety data are lacking for these compounds. Recent randomized clinical trials suggest a role for DOACs in the treatment of VTED in patients with cancer. This review will discuss the existing evidence and future perspectives on the role of DOACs in the treatment of VTE based on the current evidence about their overall efficacy and safety and the limited information in patients with cancer; in addition, we will briefly review their pharmacokinetic properties with special reference to potential interactions.

## Introduction

Venous thromboembolic disease (VTED) is a common and clinically important complication in patients with cancer [1]. Coagulation and cancer interact bidirectionally in a vicious circle in which the tumor is able to activate coagulation by producing procoagulant factors (e.g., tissue factors, cancer procoagulant proteins, microparticles, molecules of adhesion, proangiogenic factors and cytokines); these factors promote the generation of thrombin and the formation of fibrin which, in turn, favor the progression and growth of the tumor [2].

In cancer patients, factors that may be related to the increased risk of VTED could be patient-related (age, sex, race, comorbidities, immobilization, and previous history of thrombosis), cancer-related (tumor location, tumor stage, histology, and time from diagnosis) and treatment-related (periods of hospitalization and surgery, chemotherapy, antiangiogenic agents, and intravascular devices, such as central venous catheters) [3].

Currently, VTED in patients with cancer is particularly challenging, with higher rates of recurrence and major bleeding than in noncancer patients [4]. These complications are very relevant, because they contribute to mortality and morbidity. Evidence-based guidelines recommend the use of low-molecular-weight heparin (LMWH) for long-term anticoagulant treatment [5–7]. Despite clinical consensus and clinical guideline recommendations, LMWH treatment is underused [8–11], which is principally due to problems with administration, cost and patient preference.

LMWHs were developed in the late seventies and early eighties of the last century. They are obtained from the chemical or enzymatic depolymerization of heparin so that the molecules reach a molecular weight of 4000–5000 Daltons. The anticoagulant effect is due to the activation of antithrombin, which promotes the inactivation of factor Xa (and, to a lesser extent, thrombin). Compared with unfractionated heparin (UFH), which is administered intravenously, the major advantages of LMWHs are their subcutaneous administration and similar antithrombotic efficacy [12].

Direct oral anticoagulant agents (DOACs), including direct thrombin inhibitors (dabigatran) and direct factor Xa inhibitors (rivaroxaban, apixaban, edoxaban and betrixaban), are as effective as vitamin K antagonists (VKAs) for the treatment of VTED and are associated with less frequent and severe bleeding [12–14]. Three of these molecules (namely, apixaban, edoxaban and rivaroxaban) target activated factor X, and one molecule (i.e., dabigatran) is directed against activated factor II, thrombin. They are employed to prevent and treat thromboembolic events [i.e., deep vein thrombosis (DVT) or acute pulmonary embolism (PE)] or to prevent stroke and systemic embolization in cases of nonvalvular atrial fibrillation (NVAF).

They have advantages over LMWH, including a fixed-dose regimen and oral intake, predictable pharmacology and anticoagulation, and no need for regular laboratory monitoring. However, the efficacy and safety of DOACs compared with long-term LMWH have not been established, and until recently, there were limited clinical data on this topic, especially in patients suffering from cancer-associated venous thromboembolism (VTE).

In this review, we aimed to provide the most recent evidence and future perspectives regarding DOACs for the management of VTE in patients with cancer, as well as to make some recommendations based on that evidence.

## Methods

A literature search was performed in MEDLINE, The Cochrane Library, and EMBASE looking for studies published from database inception up to May 2019 on the new DOACs dabigatran, apixaban, rivaroxaban, and edoxaban. Comprehensive searches for conference abstracts were also carried out. Eligible study designs included observational analytical studies (i.e., case–control and cohort studies), randomized controlled trials, and meta-analyses of clinical trials. No language restriction was used. The eligibility criteria for being included in this review were: (1) cancer patients with a diagnosis of acute VTE; (2) reported outcomes comparing new DOACs in cancer patients; and (3) data of VTE or acute bleeding reported for up to 3 months of time.

From each study, we extracted and tabulated details on study source type, design, patients with acute VTE, mean age in years, percentage of female patients, estimated recurrent VTE risk, estimated bleeding risk (major, nonmajor and clinically relevant nonmajor bleeding), VTE events, and duration of anticoagulation.

## Role of new anticoagulants

### Dabigatran

Dabigatran is a small molecule prodrug that shows no pharmacological activity. After oral administration, dabigatran etexilate is rapidly absorbed and transformed into dabigatran by esterase-catalyzed hydrolysis in plasma and in the liver, constituting the main active substance in plasma. Dabigatran is a potent, competitive and reversible direct thrombin inhibitor that inhibits free thrombin, fibrin-attached thrombin and thrombin-induced platelet aggregation [15].

It is currently indicated for the primary prevention of venous thromboembolic episodes in adult patients undergoing scheduled total hip replacement surgery or total knee replacement surgery [16–19]. Venker et al. [20] published a meta-analysis analyzing the efficacy of 150 mg dabigatran. The relative risk (RR) of VTE was lowest for 30 mg edoxaban once daily [0.49; 95% confidence interval (CI), 0.32–0.75], 2.5 mg fondaparinux once daily (0.53; 95% CI 0.45–0.63), and 10 mg rivaroxaban once daily (0.55; 95% CI 0.46–0.66) and was highest for dabigatran (1.19; 95% CI 0.98–1.44), with a similar safety profile in terms of major/clinically significant bleeding with a HR of 1.22 (95% CI 0.89–1.67; *p* = 0.22) and 220 mg dabigatran with an HR of 1.04 (95% CI 0.87–1.24; *p* = 0.68), with major/clinically significant bleeding having an HR of 1.14 (95% CI 0.93–1.4; *p* = 0.20) compared to enoxaparin (most common dosage of 40 mg once daily). It is also indicated for the prevention of stroke and systemic embolism in adult patients with NVAF with one or more risk factors [21, 22], for the treatment of DVT and PE, and for the prevention of DVT and PE recurrence in adults [23–25]. The usual dosage is between 110 and 220 mg orally per day based on the time since surgery and with adjustments for renal function, age and concomitant medication (especially with the intake of amiodarone, verapamil and quinidine). Currently, there are no published randomized clinical trials evaluating its use in patients with VTED and cancer.

### Rivaroxaban

Rivaroxaban is a highly selective direct factor Xa inhibitor. The inhibition of factor Xa interrupts the intrinsic and extrinsic pathways of the blood coagulation cascade, inhibiting thrombin formation [26].

Rivaroxaban, coadministered with acetylsalicylic acid (ASA) alone or with ASA plus clopidogrel or ticlopidine, is indicated for the prevention of atherothrombotic events in adult patients after acute coronary syndrome (ACS) with elevated cardiac biomarkers [27]. Coadministration with ASA is indicated for the prevention of atherothrombotic events in adult patients with coronary artery disease (CAD) or symptomatic peripheral artery disease (PAD) at high risk of ischemic events [28]. It also shares the abovementioned indications of dabigatran [29–34]. The recommended dosage is 2.5 mg orally twice daily.

Regarding VTE prophylaxis in orthopedic surgeries, we note the aforementioned meta-analysis [20], with a lower RR of VTE for rivaroxaban (0.55 (95% CI 0.46–0.66; *p* < 0.001), significantly increasing the risk of clinically relevant bleeding with an HR of 1.27 (95% CI 1.01–1.59; *p* = 0.039) but not of major bleeding [HR 1.88 (95% CI 0.67–5.29; *p* = 0.23)].

Regarding VTE prophylaxis in stroke NVAF, the Cochrane Collaboration [35] published a review on the activity of factor Xa inhibitors, collecting data from 13 studies with an HR of 0.89 (95% CI 0.82–0.97) for systemic stroke/emboli, significantly reducing the risk of intracranial hemorrhage (HR 0.47, 95% CI 0.40–0.56) and major bleeding (HR 0.76, 95% CI 0.60–0.96), although the results were less robust.

### Apixaban

Apixaban is an oral, reversible, direct and highly selective inhibitor of factor Xa. It does not require antithrombin III for antithrombotic activity. Apixaban inhibits free and clot-bound factor Xa and prothrombinase activity. It has no direct effect on platelet aggregation but indirectly inhibits thrombin-induced platelet aggregation [36]. It is also indicated for the prevention of VTE in adult patients who have undergone elective hip or knee replacement surgery, for the prevention of stroke and systemic embolism in adult patients with NVAF with one or more risk factors, and for the treatment of DVT and PE, as well as the prevention of DVT and PE recurrence in adult patients [37–39].

### Edoxaban

Edoxaban is a highly selective, direct and reversible inhibitor of factor Xa that inhibits both free factor Xa and prothrombinase activity [40].

It is currently indicated for the prevention of stroke and systemic embolism in adult patients with NVAF with one or more risk factors and for the treatment of DVT and PE, as well as for the prevention of DVT and PE recurrence in adults [41–44], although its use in orthopedic surgery is not approved in Europe.

## Current evidence in cancer patients

### Treatment of venous thromboembolic disease

Real-world data studies show the use of DOACs in patients with active cancer, even before the publication of randomized controlled trials. Their results must be interpreted cautiously due to the potential selection bias inherent to observational studies of interventions. Weitz et al. [45], from the GARFIELD-VTE registry, reported 22.8% of patients with cancer receiving DOACs. Similarly, Streiff et al. [46] published a retrospective study including 707 patients treated with rivaroxaban compared with other cohorts treated with warfarin and LMWH, obtaining a trend for lower VTE recurrence rates in rivaroxaban users than in LMWH users at 6 months (13.2 vs. 17.1%; *p* = 0.060) and significantly lower recurrence rates at 12 months (16.5 vs. 22.2%; *p* = 0.030) [HR 0.72, 95% CI (0.52–0.95); *p* = 0.024]. VTE recurrence rates were also lower for rivaroxaban users than for warfarin users at 6 months (13.2 vs. 17.5%; *p* = 0.014) and 12 months (15.7 vs. 19.9%; *p* = 0.017) [HR 0.74, 95% CI (0.56–0.96); *p* = 0.028], with similar rates of major bleeding. Other studies have found that DOACs are at least as safe and effective as LMWH [47–53].

The main studies on DOACs are presented in Table 1. They included three studies conducted in the general population but reporting data of cancer patients, and 4 studies performed in cancer patients; in most cases, thrombosis recurrence was reported as the primary endpoint. The specific results of these studies are commented on in this section together with some pooled analyses and subanalyses. Phase III studies were designed to prove the efficacy of these drugs in the treatment of VTE in patients with various clinical conditions. These include the RE-COVER [23] and RE-COVER II [24] studies with dabigatran, the EINSTEIN-DVT study for DVT [54] and EINSTEIN-PE for PE [55] with rivaroxaban, and the AMPLIFY study [38] with apixaban (Table 1). The studies have the following main limitations: the small percentage of cancer patients included (5.5%) and their heterogeneity in terms of cancer type, location and stage. The results showed similar efficacy, with greater safety in favor of DOACs compared to warfarin. Subanalysis of data from the oncology population has been conducted, and thus, Prins et al. [56] published a pooled analysis of the two studies with rivaroxaban (EINSTEIN-DVT and EINSTEIN-PE) selecting the oncology subpopulation. The authors concluded that DOACs were as effective and safe as heparin combined with VKA for the treatment of VTE in cancer patients. Within the evidence-based analysis, Vedovati et al. [57] conducted a meta-analysis of randomized studies with DOACs assessing the safety and efficacy of DOACs in patients with thrombosis and cancer. Six randomized studies were included in the analysis: 2 with dabigatran, 2 with rivaroxaban, 1 with edoxaban and 1 with apixaban for a total of 1132 patients with thrombosis and active cancer. The comparator arm was LMWH followed by VKA. The recurrence of thrombosis in patients treated with DOACs was 3.9% vs. 6% for those treated with the standard of care (OR 0.63, 95% CI 0.37–1.10). In terms of safety, the incidence of episodes of major bleeding was 3.2% in patients treated with DOACs vs. 4.2% in those on standard anticoagulation [odds ratio (OR): 0.77, 95% CI 0.41–1.44].

**Table 1:** Main Studies on DOACs in the general and oncologic population

Study	*N*	Experimental arm	Primary endpoint	Cancer patients included (%)	Recurrence rate (experimental vs. standard)	Clinically relevant nonmajor bleeding/Minor bleeding rate (experimental vs. standard)	Major bleeding rate (experimental vs. standard)
EINSTEIN-DVT and PE [54, 55]	8282	Rivaroxaban (15 mg twice daily for 3 weeks, then 20 mg once daily for a total of 6 months)	Thrombosis recurrence	5.2%	5 vs. 7%	NR	2 vs. 5%
RE-COVER I-II [23, 24]	5107	Dabigatran (150 mg twice daily)	Thrombosis recurrence	6.5%	HR 0.75 (95% CI 0.2–2.8)	NR	NR
AMPLIFY [38]	5395	Apixaban 10 mg twice daily for the first 7 days, then 5 mg twice daily	Thrombosis recurrence or death due to thrombosis	3.1%	3.7 vs. 6.4%	NR	2.3 vs. 2.8%
Hokusai-VTE Cancer [58]	1050	Edoxaban (60 mg once daily)	Thrombosis recurrence or major bleeding	100%	7.9 vs. 11.3% HR 0.71 (95% CI 0.48–1.06)	97 (18.6%) vs. 73 (13.9%), HR 1.40 (1.03–1.89)/14.6 vs. 11.1% HR 1.38 (95% CI 0.98–1.94)	6.9 vs. 4% HR 1.77 (95% CI 1.03–3.04)
SELECT-D [59]	406	Rivaroxaban (15 mg twice daily for 3 weeks, then 20 mg once daily for a total of 6 months)	Thrombosis recurrence at 6 months	100%	4 vs. 11% HR 0.43 (95% CI 0.19–0.99)	6 months 4% (95% CI 2 vs. 9%) vs. 13% (95% CI 9 vs. 19%)/13 vs. 4% HR 3.76 (95% CI 1.68–8.68)	6 vs. 4% HR 1.83 (95% CI 0.68–4.96)
ADAM-VTE [60]	287	Apixaban 10 mg twice daily for the first 7 days, then 5 mg twice daily	Major bleeding	100%	0.7 vs. 6.3% HR 0.099 (95% CI 0.01–0.78)	6 months 11–14% vs. 4.3%/NR	0 vs. 1.4% HR not estimable
CARAVAGGIO [61]	1170	Apixaban 10 mg twice daily for the first 7 days, then 5 mg twice daily	Recurrent venous thromboembolism	100%	5.6 vs. 7.9% HR 0.63 (0.37–1.07) < 0.001 for noninferiority; 0.09 for superiority	9 vs. 6% HR 1.42 (0.88–2.30)	3.8 vs. 4.0% HR 0.82 (0.40–1.69); 0.60

*CI* confidence interval, *DOACs* direct oral anticoagulant agents, *DVT* deep vein thrombosis, *HR* hazard ratio *NR* not reported, *PE* pulmonary embolism, *VTE* venous thromboembolism

However, the great advance in estimating the impact of new DOACs on a population with cancer has undoubtedly been the design and publication of randomized trials in the cancer population (Table 1). Raskob et al. [58] published the results of the Hokusai-VTE Cancer study, a phase III randomized noninferiority study comparing dalteparin (at dosages of 200 IU/kg/day for 1 month followed by 150 IU/kg/day) versus oral edoxaban (60 mg orally once daily) after the first 5 days of parenteral anticoagulant. A total of 1050 patients were randomized, with a median age of 65 years, and in 63% of cases, VTE appeared at the pulmonary level in the form of PE. The primary efficacy outcome of the study was the occurrence of recurrent thrombosis, and the primary safety outcome was major or clinically relevant nonmajor bleeding. The Hokusai-VTE Cancer study was conducted in cancer patients diagnosed with DVT in the popliteal, femoral or iliac vein or inferior vena cava or in those with PE. Patients treated with bevacizumab; patients with severe renal impairment (CrCl < 30 ml/min), vena cava filters, liver disorders, or major thrombocytopenia; and those receiving treatment with 100 mg ASA, clopidogrel or NSAIDs were excluded. Treatment lasted a minimum of 6 months in both arms, with possible extension to 12 months. The primary outcome of the study was met: 12.8% of events in the edoxaban arm versus 13.5% in the dalteparin arm, with an HR of 0.97 (95% CI 0.70–1.36; *p* = 0.006 for noninferiority and *p* = 0.87 for superiority). Edoxaban also showed reduced thrombosis recurrence, but the difference did not reach statistical significance (7.9 vs. 11.3%, HR 0.71, *p* = 0.09); a statistically significantly increased incidence of major bleeding was found with edoxaban (6.9 vs. 4%, HR 1.77, *p* = 0.04) compared to LMWH, which was attributable to a greater number of gastrointestinal (GI) bleeding events with edoxaban. Young et al. [59] published the results of the SELECT-D study, a phase III comparative trial of rivaroxaban (15 mg twice daily for 3 weeks, followed by 20 mg once daily for a total of 6 months) versus dalteparin (200 IU/kg for the first month and 150 IU/kg for the following 5 months) as a treatment for VTE. The primary outcome of the study was the recurrence of DVT at 6 months. Safety was assessed by major bleeding or clinically relevant bleeding rates. Initially, the study design included a late anticoagulation phase (6–12 months) with rivaroxaban versus placebo, but it was not carried out due to low initial recruitment. A total of 406 patients were included (203 in each group), 58% of whom were patients with metastatic tumors. The rate of thrombosis recurrence was 11% for dalteparin and 4% for rivaroxaban, with an HR of 0.43 (95% CI 0.19–0.99). In terms of safety, 4% of patients on dalteparin experienced an episode of major bleeding compared to 6% in the rivaroxaban group (nonsignificant HR of 1.83), with most bleeding episodes starting in the GI tract. In an interim analysis, the inclusion of patients with esophageal and gastric cancer was stopped due to an excess of major bleeding with rivaroxaban compared to dalteparin (36 vs. 12%, respectively). In addition, clinically relevant nonmajor bleeding events were 3 times more common in the group receiving the oral anticoagulant rivaroxaban [4 vs. 13%, HR 3.69 (95% CI 1.63–8.69)].

The ADAM-VTE trial [60] was the first study comparing DOACs to LMWH in cancer patients with major bleeding as the primary endpoint. This clinical phase III randomized controlled superiority trial compared apixaban with dalteparin in 300 patients with cancer-associated VTE. In terms of the efficacy and secondary endpoint, apixaban was related to significantly lower VTE recurrence compared to dalteparin (0.7 vs. 6.3%, respectively, HR 0.26, 95% CI 0.09–0.80; *p* = 0.02). In terms of the safety and primary objective, major bleeding was not significantly different between the two arms (0 vs. 1.4%; *p* = 0.01), with a similar OS between both groups and quality of life favoring apixaban.

Recently, Li et al. [62] published a meta-analysis of studies that included cancer patients with VTE. Analyzing the two randomized studies (Hokusai-VTE Cancer and SELECT-D), edoxaban and rivaroxaban were compared with dalteparin, resulting in a tendency toward a lower recurrence in favor of DOACs (HR 0.65, 95% CI 0.42–1.01) and more frequent major bleeding in the group receiving oral anticoagulation versus LMWH (HR 1.74, 95% CI 1.05–2.88) regarding safety. Clinically relevant nonmajor bleeding showed an increased risk without reaching statistical significance (HR 2.31, CI 0.85–6.28).

However, a meta-analysis of the Hokusai-VTE Cancer and SELECT-D studies observed increased bleeding complications, especially GI bleeds, in the DOAC arm. Subgroup analysis of the Hokusai-VTE Cancer trial described a higher risk of major bleeding events in the edoxaban arm compared to in the dalteparin arm (12.7 vs. 3.6%; HR 4.0, 95% CI 1.5–10.6; *p* = 0.005), most of which were GI bleeds [63]. Soff et al. [64] recently reported a lower rate of bleeding in the DOAC arm in the Hokusai-VTE Cancer and SELECT-D trials excluding patients with GI or genitourinary malignancies.

Finally, the results of the Caravaggio trial [61], a multinational, randomized, investigator-initiated, open-label, noninferiority trial with blinded central outcome adjudication that randomly assigned consecutive patients with cancer who had symptomatic or incidental acute proximal DVT or PE to receive oral apixaban (at a dosage of 10 mg twice daily for the first 7 days, followed by 5 mg twice daily) or subcutaneous dalteparin (at a dosage of 200 IU per kilogram of body weight once daily for the first month, followed by 150 IU per kilogram once daily), have been published. The treatments were administered for 6 months. The primary outcome, recurrent VTE, was objectively confirmed during the trial period. The principal safety outcome was major bleeding. It randomized 1170 patients, and it is the largest trial ever conducted on cancer-associated thrombosis treatment. Patients with primary brain tumors, intracerebral metastases, or acute leukemia were not eligible. Recurrent VTE occurred in 32 of 576 patients (5.6%) in the apixaban group and in 46 of 579 patients (7.9%) in the dalteparin group (HR 0.63, 95% CI 0.37–1.07; *p* < 0.001 for noninferiority). Major bleeding occurred in 22 patients (3.8%) in the apixaban group and in 23 patients (4.0%) in the dalteparin group (HR 0.82, 95% CI 0.40–1.69; *p* = 0.60). Additionally, major GI bleeding was similar between arms, 1.9% in the apixaban arm versus 1.7% in the dalteparin arm. No difference in mortality was observed, with a death rate of any cause at 7 months of 3.4% for apixaban compared to 26.4% for dalteparin. However, event-free survival, defined as the absence of recurrent VTE, major bleeding or death, was superior for the apixaban arm: 73.3% versus 68.6% (HR 1.36, 95% CI 1.05–1.76). The authors concluded that oral apixaban was noninferior to subcutaneous dalteparin for the treatment of cancer-associated VTED, without an increased risk of major bleeding.

### Treatment with direct oral anticoagulants in cancer patients with upper extremity deep vein thrombosis (UEDVT)

Globally, UEDVT accounts for approximately 10% of all DVTs and can trigger both PE and postphlebitic syndromes in the arm. The prevalence of UEDVT is increasing due to the increased use of central venous catheters of the port-a-cath type, Hickman type, etc. in patients with cancer and chronic diseases [65, 66].

The effectiveness and safety of new DOACs is being studied in various studies in this group of patients, and the limitations are similar to those of lower limb DVT and PE in small series, with little representation of patients with cancer and heterogeneous populations in terms of tumor type and stage. The Swedish registry AuriculA [65] includes 55 patients with UEDVT treated with DOACs [46 patients with rivaroxaban, 7 with apixaban and 2 with dabigatran]. Of the 55 patients, 10 (18%) had a malignancy. After 6 months of DOAC treatment, there was 1 case of recurrence during treatment (2%) and 2 cases of recurrences after the end of the treatment period (4%). Only 1 patient (2%) experienced a nonmajor bleeding episode.

### Rivaroxaban and apixaban in the initial treatment of acute venous thromboembolism of atypical location (VTE-AL) in cancer patients

Approximately 4% of VTE cases include atypical locations, such as splenic, renal, gonadal and cerebral vein territories [67–71]. These locations, although less frequent, are relevant in clinical practice, as they are often associated with neoplastic processes and require more specialized knowledge to decide the need for anticoagulant treatment. The main problem with apixaban and rivaroxaban trials is that they include mostly patients with confirmed PE and/or proximal DVT of the lower limbs and exclude atypical locations [38, 54, 55].

There are few studies that assess the use of DOACs in atypical locations, which makes it difficult to draw conclusions about the use of DOACs in these locations and in cancer patients.

Between March 2013 and February 2017, the Mayo Clinic created a registry of VTE patients prospectively followed to assess the rates of recurrence, major bleeding, clinically relevant nonmajor bleeding and survival [72]. Portal, mesenteric, splenic, hepatic, renal, ovarian or brain venous sinus territories were defined as atypical locations. Out of the 36 patients identified in the VTE-AL registry receiving DOACs, 19 (52.8%) had a malignancy, 13 (36.1%) were on chemotherapy, and 11 (30.6%) had undergone recent surgery. Of the 23 patients with VTE-AL who were on enoxaparin, 22 (95.7%) had a malignancy, 12 (52.2%) were on chemotherapy, and 6 (26.1%) had undergone a recent surgery. The percentage of patients with malignancy and VTE-AL who had received DOACs was significantly lower than the percentage of patients with malignancy and VTE who had received enoxaparin (52.8 vs. 95.7%; *p* < 0.001), a fact that already implies a selection bias that, together with the recording characteristics of the study, limits the possibility of drawing conclusions. The authors observed higher mortality rates in patients with VTE-AL treated with DOACs (21.5 per 100 person-years; 95% CI 7.9–46.7) compared to those with VTE (8.3; 95% CI 5.4–12.2; *p* = 0.03). Mortality was higher in the VTE-AL group on DOACs than in the VTE-TL group [21.45 (95% CI 7.87–46.69) vs. 8.26 (95% CI 5.35, 12.20); *p* = 0.03]. The authors concluded that neither the recurrence rate nor the bleeding from rivaroxaban and apixaban in the treatment of VTE-AL were different from the recurrence or bleeding rates of DOAC-treated VTE or enoxaparin-treated VTE-AL.

## Primary prophylaxis of VTE

Primary thromboprophylaxis is recommended in hospitalized patients with active cancers and is not recommended in the routine care of ambulatory patients treated with chemotherapy [73, 74]. This indication should be considered for select high-risk patients considering the clinical and tumor characteristics [75–80] and the clinical prediction scores, such as Khorana et al. and others [77, 81, 82] who modify the original score, including the Protecht score [83] and Vienna score [84].

Some recently published clinical trials have reported results regarding the utility of DOACs in this setting (Table 2). The AVERT trial [85], a randomized, placebo-controlled, double-blind clinical trial, compared 6 months of 2.5 mg apixaban twice daily with placebo in 574 patients with a Khorana score ≥ 2. The primary efficacy outcome rate was lower in the apixaban group than in the placebo group (4.2 vs. 10.2%; HR 0.41, 95% CI 0.26–0.65; *p* < 0.001; 17 patients needed to be treated to avoid 1 episode of VTE). Major bleeding was higher in the apixaban group, except when the analysis was limited to the treatment period (2.1 vs. 1.1%; HR 1.89, 95% CI 0.39–9.24). No fatal bleeds occurred, and no difference in nonmajor bleeding or mortality was observed. The CASSINI trial [86] was a double-blind, randomized, placebo-control multicenter study that compared rivaroxaban with placebo in 841 ambulatory patients with a Khorana score ≥ 2. The primary endpoint (objectively confirmed VTE and death from VTE assessed up to day 180) occurred in 6% of patients in the rivaroxaban group and in 8.8% of patients in the placebo group (HR 0.66; 95% CI 0.40–1.09; *p* = 0.10). During the on-treatment period, rivaroxaban significantly reduced the primary endpoint of VTE or VTE-related death compared with placebo (2.62 vs. 6.41%; HR 0.40, 95% CI 0.20–0.80; *p* = 0.007; with a number needed to treat of 26 patients). No difference was observed between groups during the analysis of the full observation period. The rates of major bleeding were similar in both groups (1.98 vs. 0.99%; HR 1.96, 95% CI 0.59–6.49; *p* = 0.265).

**Table 2:** Randomized clinical trials assessing the efficacy and safety of DOACs in the prophylaxis of cancer-associated thrombosis

Characteristic/TRIAL	CASSINI [86] (*n* = 841)	AVERT [87] (*n* = 574)
Trial hypothesis	Superiority	Superiority
Arms	Rivaroxaban (10 mg once daily) vs. placebo	Apixaban (2.5 mg twice daily) vs. placebo
Inclusion criteria	Ambulatory patients with cancer initiating chemotherapy at intermediate-to-high risk for VTE (Khorana score ≥ 2)	Ambulatory patients with cancer initiating chemotherapy at intermediate-to-high risk for VTE (Khorana score ≥ 2)
Cancers included	Ambulatory outpatients with a solid tumor or lymphoma (excluded if they had a primary brain tumor or known brain metastases)	Newly diagnosed cancer or progression of known cancer after complete or partial remission. Excluded basal cell or squamous cell skin carcinoma, acute leukemia or myeloproliferative neoplasms
Primary efficacy outcome	Objectively confirmed VTE and death from VTE, assessed up to day 180	First episode of objectively documented major VTE
Primary outcome results	6% in the rivaroxaban group vs. 8.8% in the placebo group (HR 0.66; 95% CI 0.40–1.09; *p* = 0.10). IPA 2.62 vs. 6.41% (HR 0.40, 95% CI 0.20–0.80, *p* = 0.007)	4.2% (apixaban) vs. 10.2% (placebo) (HR 0.41; 95% CI 0.26–0.65; *p* < 0.001)
Safety outcome: MB	1.98 vs. 0.99%, HR 1.96, 95% CI 0.59–6.49; *p* = 0.265	During treatment period: MB2, 1% (apixaban) vs. 1.1% (placebo) (HR 1.89; 95% CI 0.39–9.24) ITT analysis: MB 3.5% (apixaban) vs. placebo (HR2; 95% CI 0.39–9.24)

*CI* confidence interval, *DOACs* direct oral anticoagulant agents, *IPA* prespecified intervention period, *ITT* intention-to-treat-analysis, *MB* major bleeding, *VTE* venous thromboembolism

The results of these studies suggest that the included DOACs (apixaban and rivaroxaban) may reduce the rate of VTE as primary thromboprophylaxis in high-risk patients with an acceptable safety profile. Some limitations of these studies should be considered for future randomized trials, without avoiding the fact of the different inclusion and exclusion criteria of both studies as well as the use of expanded Khorana scores in the AVERT trial compared with the original Khorana scores in the CASSINI trial, as they provide specific knowledge on the risk and benefit of thromboprophylaxis in specific cancer types, the correct evaluation of bleeding risk considering that both studies excluded patients at a high risk of bleeding, the duration of primary thromboprophylaxis in ambulatory cancer patients receiving chemotherapy with Khorana scores not validated for the risk stratification of patients at 6 months postchemotherapy initiation period, the usefulness of serial VTE screening image techniques in the care of patients and the consideration of the complexity of cancer patients and the multiple expectations that should be influenced before any intervention.

### Absorption, metabolism and potential interactions

Due to oral administration and particularities in absorption, some aspects of DOAC metabolism and excretion may limit the use of these drugs. Considering absorption, rivaroxaban moderately interferes with food, requiring an intake of 15–20 mg. A minimal effect was observed with edoxaban, and no effect was observed with dabigatran and apixaban, although a rate of dyspepsia of 5–10% was observed with dabigatran. Some authors have suggested fluoropyrimidine-induced GI damage that might reduce the plasma concentration of dabigatran by impairing absorption [87].

Regarding metabolism, apixaban, rivaroxaban and edoxaban, as well as substrates of cytochrome P450 isoenzyme 3A4 to various degrees, with minimal interaction of edoxaban (< 4% excretion) and moderate interaction of apixaban (25%), are also substrates of P-glycoprotein (P-gp), and the prodrug dabigatran etexilate is also a P-gp substrate. Strong inducers significantly decreased plasma DOAC levels, and strong inhibitors increased disease levels [88, 89]. Some anticancer drugs with potential interactions include tyrosine kinase inhibitors, antimitotic microtubules and some immune-modulating agents, including glucocorticoids [89]. Other interactions include cyclosporine increasing plasma levels of edoxaban [90] and some groups with potential interactions, such as anthracyclines, topoisomerase inhibitors, and alkylating and hormonal agents [89].

Other clinical situations that can cause interactions with DOACs are chemotherapy-induced nausea and vomiting [4] and alterations in renal function in which dabigatran and edoxaban have a renal clearance of 80 and 50%, respectively; thus, careful dosing and drug labeling should be considered in patients with renal failure [91–94] and thrombocytopenia, considering anticoagulation in patients with < 25,000 platelets/µl who are at lower risk for recurrent VTE.

### Upcoming studies

There are ongoing studies evaluating DOACs that will provide further evidence for the use of these agents in cancer patients and will help to clarify remaining questions. The CASTA-DIVA (NCT02746185) [37] study (rivaroxaban vs. dalteparin) for the treatment of VTE in patients with cancer and high risk of VTE recurrence has primary outcomes of recurrent VTE, major bleeding and clinically relevant nonmajor bleeding and mortality; the CANVAS trial (NCT027440923)36 (DOAC vs. LMWH or LMWH + VKA) has a primary outcome of cumulative VTE recurrence; the APICAT trial compares the efficacy and safety of two doses of apixaban for the extended treatment of VTE in cancer patients (breast, prostate and colon-rectum); the EVE-Extended study compares the safety of two doses of apixaban for the extended treatment of cancer patients with VTE; and other studies such as COSIMO37 (NCT027426239), a prospective cohort study (patient-reported outcomes with rivaroxaban: a noninterventional study), and Conko-011 (NCT02583191) comparing rivaroxaban versus LMWH (patient-reported treatment satisfaction).

## Recommendations

Based on the information reviewed, the following recommendations can be made:

1. According to the evidence described in this review, we cannot make specific recommendations for prophylaxis of VTE in hospitalized medical and surgical cancer patients.
2. Considering VTE prophylaxis in ambulatory cancer patients during systemic therapy assessment, the results of the AVERT and CASSINI trials suggest that, although routine thromboprophylaxis is not recommended (level of evidence: grade 1B), apixaban and rivaroxaban may reduce the rate of VTE via primary thromboprophylaxis in high-risk patients with an acceptable safety profile, such as in patients with advanced pancreatic cancer; patients with a Khorana score ≥ 2 or patients considered high-risk based on a validated risk assessment model; patients who have initiated systemic therapy and no contraindications to anticoagulation; and patients with a low risk of bleeding. There is no consensus about the dose and duration of the thromboprophylaxis; it is suggested that it should last at least 12 weeks after the initiation a new systemic therapy. A specific drug–drug interaction assessment must be done.
3. In the treatment of VTE in cancer patients, although LMWH at a body weight-adjusted dose is the drug of choice for the initial treatment (level of evidence: grade 1B), DOACs can be an alternative in the treatment of these patients, especially in patients at low risk of bleeding (increased risk of GI and probably genitourinary bleeding), and they have no significant drug–drug interactions (level of evidence: grade 1A). Edoxaban and rivaroxaban are equally or more effective than dalteparin for the prevention of VTE recurrence but confer a higher risk for major bleeding, especially in GI cancers. The recent publication of the results of the Caravaggio trial presents apixaban as a good alternative in this group of patients considering efficacy and no increased risk of major bleeding (including GI major bleeding).
4. It is difficult to reach a conclusion for the treatment of VTE with DOACs in UEDVT, an atypical location, since these events were not included in the largest randomized clinical trials of DOACs (neither in analyses of cancer patients nor in subanalyses provided for this outcome).
5. A switch to DOACs can be a good alternative in the treatment of VTE recurrence (level of evidence: grade 2B). If recurrence occurs while under treatment with DOACs, a switch to LMWH should be considered; alternatively, DOAC dose escalation should be considered if an infratherapeutic dose was used (level of evidence: grade 3C).
6. DOACs have emerged as an attractive alternative to LMWH in the treatment of VTE in cancer patients. All benefits and risks of each of these agents should be taken into consideration and evaluated in individual patients based on their risk–benefit profile, comorbidities and probable adherence to treatment. Furthermore, the risk of VTE also varies throughout the clinical course of cancer, and for this reason, the choice of anticoagulant should be considered in accordance with the current presence of robust data supporting the use of DOACs for the treatment and prevention of VTE in selected patients. GI and urothelial cancer patients require extreme caution due to the high rates of major bleeding. Even with the publication of new and ongoing studies, the evidence for DOAC treatment in cancer-associated VTE has limitations that should prompt further research: the patient selection for prior clinical trials may not reflect the entire patient population, there is little evidence on the optimal duration of anticoagulation, and there are doubts concerning which patients are most likely to benefit from DOAC therapy.
